# *Calotropis procera* Root Extract Has the Capability to Combat Free Radical Mediated Damage

**DOI:** 10.1155/2013/691372

**Published:** 2013-10-09

**Authors:** Shashank Kumar, Ashutosh Gupta, Abhay K. Pandey

**Affiliations:** ^1^Department of Biochemistry, University of Allahabad, Allahabad 211002, India; ^2^Department of Biotechnology, Sam Higginbottom Institute of Agriculture, Technology and Sciences, Allahabad 211007, India

## Abstract

The present study reports the antioxidant and membrane protective activities of *Calotropis procera *aqueous root extract using several *in vitro *assays along with the determination of phenolic as well as flavonoid contents. Total phenol and flavonoid contents in extract were 15.67 ± 1.52 mg propyl gallate equivalent/g and 1.62 ± 0.05 mg quercetin equivalent/g, respectively. UV-visual spectroscopic scanning of the extract indicated the presence of glycoside-linked tannins or flavonoids. The extract exhibited appreciable reducing power signifying hydrogen donating potential. DPPH radical scavenging assay revealed substantial free radical scavenging activity (42–90%) in the extracts. Concentration dependent response was observed in the metal ion chelating activity (16–95%). Extracts also provided protection against iron induced lipid peroxidation in rat tissue (liver, brain, and kidney) homogenates. Comparatively better protective efficacy against peroxidative damage was observed in liver (71%) followed by kidney (65%) and brain (60%) tissues. Positive correlation (*r* ^2^ = 0.756) was observed between DPPH free radical scavenging activity and reducing power of extract. Similarly strong positive correlation (*r* ^2^ ≈ 0.756) was observed between metal ion chelating ability and percentage lipid peroxidation inhibition in different tissues. The study demonstrated considerable protective efficacy in *C. procera *root aqueous extracts against free radical and metal ion mediated oxidative damage.

## 1. Introduction

In cells, free radicals are generated due to redox process when oxygen is utilized to generate energy. The free radicals constitute a group of reactive oxygen species (ROS) such as hydroxyl radical, hydrogen peroxide, and super oxide anion [1]. ROS are beneficial for cellular responses and immune function at lower concentrations, while at higher levels they are harmful to the body. Oxidative stress is caused by the excess free radical generation in the body which is capable of damaging cell structure including lipids, proteins, and DNA [2]. Overload of free radical plays a major role in the development of degenerative and chronic ailments such as rheumatoid arthritis, autoimmune disorders, cataract, aging, cancer, and cardiovascular and neurodegenerative diseases [3, 4]. Antioxidants being free radical scavengers minimize the damage caused by ROS and thereby enhance the immune defense and lower the risk of degenerative disorders [2]. Antioxidants are either naturally produced *in situ* or supplied through foods and supplements [5]. Flavonoids and phenolic compounds of natural origin have been shown to act as effective antioxidant as well as antibacterial agents [6, 7]. Lipid peroxidation causes damage to unsaturated fatty acids and thereby decreases membrane fluidity which ultimately leads to many pathological events in the body [8]. Antioxidants also protect membranes from peroxidative damage through their metal ion chelating and radical scavenging capabilities [6].

*Calotropis procera *is a wild growing plant that belongs to family Asclepiadaceae. Worldwide, it is known by various names such as swallowwort, dead sea apple, sodom apple or milk weed. In India, the plant is known as madar in Hindi, orka in Oriya, and alarka in Sanskrit [9]. In the traditional Indian medicinal system, it has been used for the treatment of leprosy, ulcers, tumors, piles, and diseases of spleen, liver and abdomen [10–12]. The plant is also known for its toxic properties that include iridocyclitis, dermatitis and acts like a poison and produces lethal effects [13]. The aqueous flower extract has been shown to possess analgesic, antipyretic, and anti-inflammatory activities [14, 15]. Decoction of the aerial parts of plant exhibits neuromuscular blocking activity [16]. The ethanolic extracts of the different parts specially flower and bud extracts have been reported to possess an antimalarial activity [17]. The chloroform extract of seeds displays antimicrobial activity [18]. Most of the published reports on the biological activity of *C. procera* are related with the latex and some organic extracts derived from the aerial parts. Not much work is on record related with the biochemical activities of the root. Therefore the present study was undertaken to determine phenolic as well as flavonoid contents and to investigate the antioxidant and membrane protective activities of *C. procera* aqueous root extract by using several *in vitro* assays.

## 2. Materials and Methods

### 2.1. Plant Material

*C. procera* roots were collected October 2012 from the Science Faculty Campus, University of Allahabad and identified by an expert in Botany Department, University of Allahabad, Allahabad, India. The voucher specimen has been kept in our department (AU/BCH/AKP/07). The roots were shade dried at room temperature for 10–15 days and ground into fine powder in a mixer grinder.

### 2.2. Preparation of Extract

A fifty gram powdered sample was extracted with water (AQ) in Soxhlet apparatus for 8 h [19, 20]. The extract was centrifuged at 4000 rpm for 10 min at 4°C and filtered using Whatman 1 filter paper (pore size 11 *μ*m). The solvent was removed completely under reduced pressure. The percentage yield of aqueous extract was 19.43%. The dried residues were dissolved in distilled water for the determination of phytoconstituents and antioxidant and antilipid peroxidation activities of the extract.

### 2.3. UV-Visible Spectroscopic Scanning

*C. procera* root aqueous extract was dissolved in distilled water (10 *μ*g/mL), filtered and scanned in the spectrophotometer at medium speed in UV-Visible range for specific absorbance identification that might appear for its chemical constituents [21]. The characteristics of molecules to absorb radiations under specific wavelengths were scanned in the entire range of 190–750 nm. 

### 2.4. Determination of Total Phenolics

The amount of total phenol in *C. procera* extract was determined according to the protocols [22, 23]. The extract (20 mg) was dissolved in 1 mL distilled water for phenol estimation. Samples (0.2 mL) were diluted to 3 mL with water. Small amount (0.5 mL) of twofold-diluted Folin-Ciocalteu reagent was added, and the contents were mixed. After 3 min, 2 mL of 20% Na_2_CO_3_ solution was added, and the tubes were placed in boiling water bath for one min followed by rapid cooling. The absorbance was measured at 650 nm against a reagent blank using spectrophotometer (Visiscan-167, Systronics). The calibration curve was prepared using tubes containing 0.2 mL standard propyl gallate solution having different concentrations (10–50 *μ*g per tube) and colour was developed as described above. The concentration of phenols in the test samples was expressed as mg propyl gallate equivalents/g material (mg PGE/g). The estimation of phenolics in the extract fractions was carried out in triplicate, and the results were expressed as mean ± SD.

### 2.5. Quantitative Determination of Total Flavonoid Content

Aluminum chloride colorimetric method [24] was used for the determination of flavonoids in root aqueous extract. Small amount (0.2 mL) of extract in distilled water was mixed with 1.8 mL of methanol, 0.1 mL of 10% aluminum chloride, 0.1 mL of 1 M potassium acetate and 2.8 mL of distilled water. Tubes were incubated at room temperature for 30 min, and then the absorbance of the reaction mixture was measured at 415 nm. The calibration curve was prepared with quercetin solution (1 mg/mL in methanol). Different volumes containing 20–200 *μ*g quercetin were taken in different tubes, and the volume was raised to 1.8 mL with methanol followed by the addition of 0.2 mL distilled water. Rest of the procedure was the same as described above. The amount of flavonoids in the test samples was expressed as mg quercetin equivalent/g of sample (mg QE/g). Experiments were performed in triplicate, and the results were expressed as mean ± SD.

### 2.6. Reducing Power Assay

The reducing power of *C. procera* root aqueous extract was determined by the methods [25, 26]. One mL aliquots of extracts (1–10 mg/mL) prepared in distilled water were taken in test tubes. To each test tube 2.5 mL of phosphate buffer (0.2 M, pH 6.6) and 2.5 mL of 1% potassium hexacyanoferrate [K_3_Fe (CN)_6_] were added, and contents were mixed. Tubes were then incubated at 50°C in a water bath for 20 min. The reaction was stopped by adding 2.5 mL of 10% trichloroacetic acid (TCA) solution and then centrifuged at 4000 g for 10 min. One mL of the supernatant was mixed with 1 mL of distilled water and 0.5 mL of ferric chloride solution (0.1%, w/v) and kept at room temperature for 2 min. The absorbance was measured at 700 nm. The BHA (butylated hydroxyanisole) was used as positive control for comparison. All the tests were run in triplicate. Results are reported as mean ± SD. Higher absorbance indicated the higher reducing power.

### 2.7. Metal Ion Chelating Activity

The chelation of ferrous ions by the *C. procera* root extract was estimated by the method [27] as modified by us [8]. Modification included dissolution of extracts in distilled water instead of methanol. Briefly, the extract samples (200 *μ*L) containing different amounts (0.5–4 mg) were added to a solution of 2 mM/L ferric chloride (0.05 mL). The reaction was initiated by the addition of 5 mM ferrozine (0.2 mL), and the mixture was shaken vigorously and left standing at room temperature for 10 min. Absorbance of the solution was then measured spectrophotometrically at 562 nm. BHT (butylated hydroxytoluene) was used as positive control for comparison. The inhibition percentage of ferrozine—Fe_2_^+^ complex formation was calculated by the formula given below:

(1) $$\begin{array}{c} \%\;\text{metal}\;\text{ion}\;\text{chelating}\;\text{ability}=\left[\frac{\left(A_{0}-A_{1}\right)}{A_{0}}\right]\;\times 100, \end{array}$$

where *A*_0_ is the absorbance of control and *A*_1_ is the absorbance in the presence of the sample/standard compounds. The results were expressed as mean ± SD of three replicates.

### 2.8. DPPH Radical Scavenging Activity

The free radical scavenging activity of the extract was measured *in vitro* by 1,1-diphenyl-2-picrlhydrazyl (DPPH) assay [28]. Three milliliters of 0.1 mM DPPH solution prepared in methanol was added to 1 mL of the test extracts (1–10 mg/mL) dissolved in distilled water. The content was mixed and allowed to stand at room temperature for 30 min in the dark. The reduction of DPPH free radical was measured by recording the absorbance at 517 nm. BHA and BHT were used for comparison. The percentage scavenging activities (% inhibition) at different concentrations of the extracts fractions were calculated using the following formula.

(2) $$\begin{array}{c} I\left(\%\right)=\left[\frac{\left(A_{C}-A_{S}\right)}{A_{C}}\right]\;\times 100, \end{array}$$

where *I* is inhibition, *A*_*C*_ and *A*_*S*_ are the absorbance values of the control and the sample respectively. Three replicates were made for each sample and results were expressed as mean ± SD.

### 2.9. Lipid Peroxidation Inhibition (LPOI) Assay

The lipid peroxidation inhibition by the *C. procera* root extract was measured by the method of Halliwell and Gutteridge [29]. Tissue homogenate (10% w/v) was prepared by homogenizing fresh normal albino rat liver, brain and kidney tissues using phosphate buffer saline, pH 7.4. The homogenate was centrifuged at 3000 rpm for 15 min, and clear supernatant were taken for analysis. 100 *μ*L extract of different concentration dissolved in distilled water was taken in test tubes and evaporated to dryness followed by addition of 1 mL of 0.15 M potassium chloride and 0.5 mL of tissue homogenate. Peroxidation was initiated by adding 0.2 mM ferric chloride (100 *μ*L). The tubes were incubated at 37°C for 30 min. The reaction was stopped by adding 2 mL of ice-cold hydrochloric acid (0.25 N) containing 15% TCA, 0.38% thiobarbituric acid (TBA) and 0.5% BHT. The reaction mixture was incubated at 80°C for 1 h. The samples were cooled and centrifuged and the absorbance of the pink supernatant was measured at 532 nm. BHA was used as control for comparison. A similar experiment was performed in the absence of the extract and standard to determine the amount of lipid which served as control. All analyses were carried out in triplicate, and results were expressed as mean ± SD. The percentage of lipid peroxidation inhibition (% LPOI) was calculated by using the following formula:

(3) $$\begin{array}{c} \text{LPOI}\;\left(\%\right)=\left[\frac{\left(A_{0}-A_{1}\right)}{A_{0}}\right]\;\times 100, \end{array}$$

where *A*_0_ is the absorbance of control and *A*_1_ is the absorbance of the standards or samples.

### 2.10. Statistical Analysis

All experiments were carried out in triplicate and data were expressed as mean ± standard deviation (SD). The plots were prepared using Microsoft excel and Graph pad Prism software. Data were analyzed using one-way and two-way ANOVA, and the values of *P* < 0.05 were considered as statistically significant. 

## 3. Results

### 3.1. UV-Visible Spectroscopic Scanning

**Figure 1:**
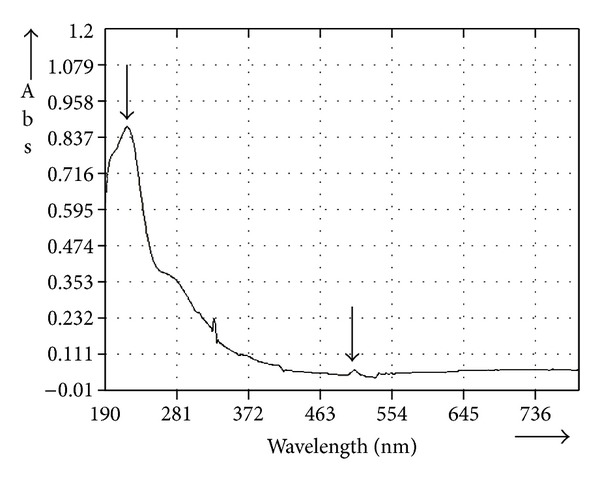
UV-Visible spectroscopic scan of *C. procera* root aqueous extract.

The characteristics of molecules to absorb radiations under specific wavelengths were scanned in the entire range of 190–750 nm. The UV-Visible scan of *C. procera* root aqueous extract is shown in Figure 1. One major absorbance peak was observed at 220 nm, while an other minor peak was observed at 510 nm. The major peak indicated presence of glycoside-linked tannins or flavonoids in the test extract [30] while minor peak at 510 nm indicated the presence of a small amount of anthocyanins [31].

### 3.2. Total Phenolic and Flavonoid Contents

Total phenolic and flavonoid contents in *C. procera* root aqueous extract were found to be 15.67 ± 1.52 mg PGE/g and 1.62 ± 0.05 mg QE/g, respectively.

### 3.3. Reducing Power Assay

**Figure 2:**
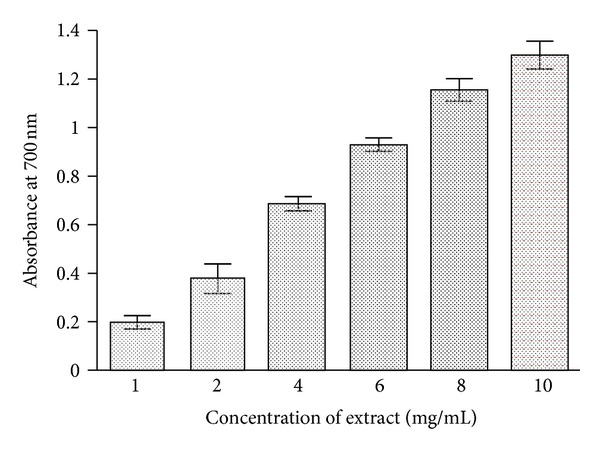
The reducing power of *C. procera* root aqueous extract. The extracts were prepared in water as described in Section 2. Butylated hydroxyanisole (BHA) was used as standard antioxidant for comparison. Six concentrations (1, 2, 4, 6, 8, and 10 mg/mL) have been used to evaluate the antioxidant activity of extracts as mentioned in Section 2. The results are expressed as mean ± SD of three replicates (*P* < 0.05).

Considerable reducing power was observed in root extract, and results are shown in Figure 2. Higher absorbance values indicated higher reducing power. The reducing power of the extract increased with increasing the concentration (1–10 mg/mL) of extract exhibiting dose dependent response (0.198–1.299). At the lowest test concentration (1 mg/mL), BHA exhibited considerable reducing power (*A* = 1.560, not shown in figure). At higher concentrations, reducing power of extract was comparable to that of BHA.

### 3.4. DPPH Radical Scavenging Potential

**Figure 3:**
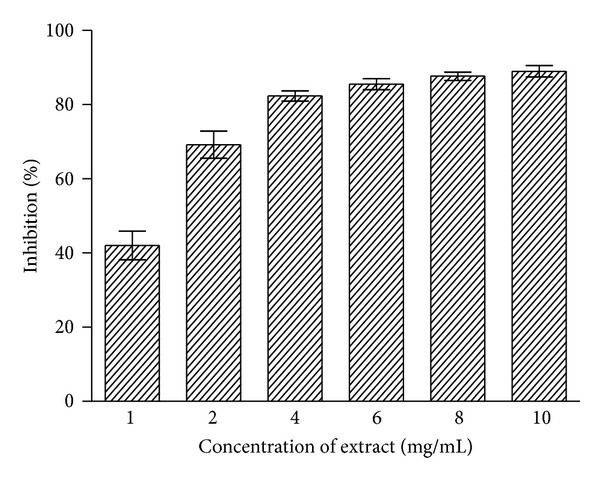
Free radical scavenging activity of *C. procera* root aqueous extracts by DPPH assay. Phytochemicals present in root were extracted with water, and radical scavenging activity of the extracts was measured at six different concentrations as described in Section 2. The results are expressed as mean ± SD of three replicates (*P* < 0.05).

Free radical scavenging potentials of *C. procera* root aqueous extract at different concentrations (1–10 mg/mL) were measured by the DPPH radical scavenging assay, and the results are shown in Figure 3. The degree of discoloration indicates the scavenging potentials of the extracts. Considerable scavenging potential (42–82%) was found at lower concentrations (up to 4 mg/mL) of the extract. Further increase in extract concentration produced little enhancement in activity (about 90%). both BHA and BHT exhibited appreciable radical scavenging activities (about 95%) at lower concentration (0.5 mg/mL).

### 3.5. Metal Ion Chelating Ability

**Figure 4:**
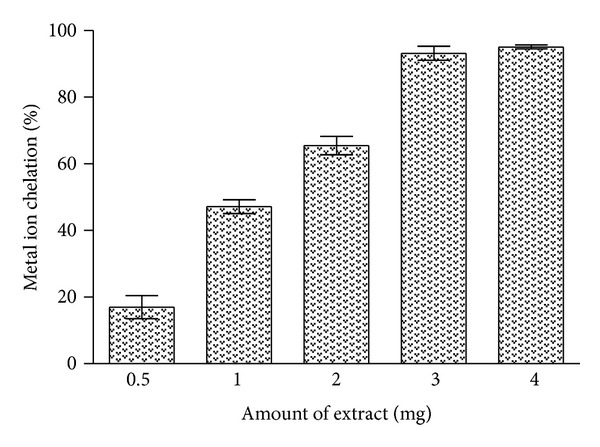
Metal ion chelating activity of *C. procera* root aqueous extract. Phytochemicals present in the root sample were extracted with water as described in Section 2. Metal ion chelating ability of extracts was measured in the presence of different amounts and absorbance was recorded at 562 nm. The results are expressed as mean ± SD of three replicates (*P* < 0.05).

Dose dependent effect in metal ion chelating activity was observed with increasing the amount of *C. procera* root aqueous extract in the reaction mixture, and activity was found in the range of 16–95% as shown in Figure 4. The degree of decrease in coloration indicates the chelation potentials of the extract. Appreciable increase in chelating activity was observed in presence of 0.5–3 mg of extract. Further increase in extract concentration did not produce noticeable increment in chelating potential. At 3 mg concentration the extract exhibited about 93% chelating ability. BHT (0.5 mg) produced about 90% chelating activity.

### 3.6. Lipid Oeroxidation Inhibition Activity

**Figure 5:**
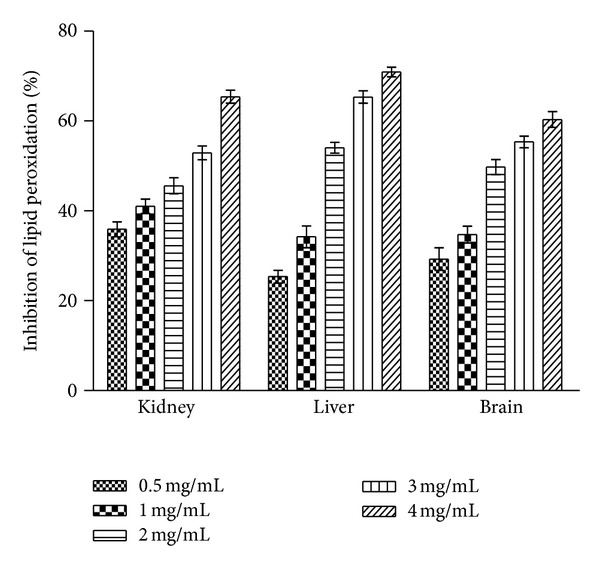
Inhibition of lipid peroxidation (% LPOI) in rat tissue (kidney, liver, brain) homogenates by *C. procera *root aqueous extracts at different concentrations (0.5–4 mg/mL). Phytochemicals present in root were extracted with water, and % LPOI was measured as described in Section 2. BHA (2 mg/mL) was used as standard for comparison which exhibited about 85% LPOI against all tissues. The results are expressed as mean ± SD of three replicates (*P* < 0.05).

Root extract of *C. procera* showed concentration dependent antilipid peroxidation activity *in vitro* (Figure 5) and exhibited capability of protecting tissue from peroxidative damage. In brain and liver homogenate test the extract exhibited significant dose dependent protective efficacy against lipid peroxidation activity. Aqueous extract significantly inhibited Fe_2_^+^-induced lipid peroxidation in different organ tissues indicating lipoprotective response. The % LPOI activity in the presence of the highest test concentration of extract was about 71%, 65%, and 60% for liver, kidney and brain tissue homogenates (Figure 5), respectively. At the lowest concentration of the extract, about 30% LPOI activity was observed. Standard antioxidant BHA (2 mg/mL) exhibited about 85% LPOI against all tissues.

## 4. Discussion

Phytochemicals have the potential to modulate human metabolism in a manner beneficial for the prevention of chronic and degenerative diseases [1, 26, 32]. Chemically, phenolics are polyphenols which are important secondary metabolites present in plants having various beneficial effects including antioxidant potential. Plant-derived antioxidants such as lignans, tannins, stilbenes, quinones, coumarins, xanthones, phenolic acids, flavones, flavonols, catechins, anthocyanins, and proanthocyanidins have the potential to delay or provide protection to living organisms from the damage caused by uncontrolled production of ROS and the concomitant lipid peroxidation, protein damage, and DNA strand breaking because of their redox properties, which allow them to act as hydrogen donors, reducing agents, and free radical scavengers [33–35]. On the basis of carbon skeleton, polyphenols are classified as flavonoids and phenolic acids. Flavonoids are secondary metabolites characterized by flavan nucleus, that is, 2-phenyl-benzo-*γ*-pyrane nucleus consisting of two benzene rings linked through a heterocyclic pyran ring [36, 37]. Phenolics and flavonoids have been reported to exert anti-inflammatory, antiviral, antibacterial, antiallergic, antitumour, lipid peroxidation and platelet aggregation inhibitor activities [38, 39]. They exert these effects as antioxidants, chelators of divalent cation, and free radical scavengers [40, 41].

Considerable amounts of phenolics (15.67 ± 1.52 mg PGE/g) and flavonoids (1.62 ± 0.05 mg QE/g) were found to be present in *C. procera* root aqueous extract. Biochemical activities studied *in vitro* in the present work might be attributed to the presence of these components. UV-Visible spectroscopic scan of the test extract showed a prominent peak at 220 nm (Figure 1) indicating the presence of glycoside-linked tannins or flavonoids [30] which further substantiates that most of the activities observed during study are because of the presence of phenolics and flavonoids. 

Fe^3+^ to Fe^2+^ transformation in the presence of plant extract has been used for the measurement of the reductive ability of plant extract as described by Oyaizu [25]. *C. procera* root extract possessed considerable reductive ability (Figure 2). Earlier authors have observed a direct correlation between antioxidant activity and reducing power of plant extracts. Reducing ability of extracts is due to presence of reductones [42, 43]. They exert antioxidant action by donating a hydrogen atom that breaks the free radical chain [44]. Study shows that test extract has appreciable ability to convert Fe^3+^ into Fe^2+^, that is, capability to donate hydrogen atom, a property of antioxidants.

**Figure 6:**
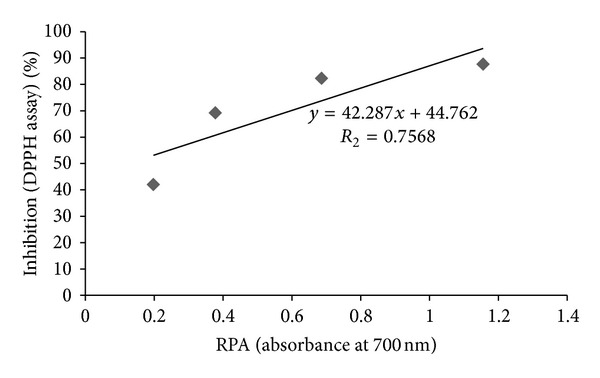
Relationship between DPPH free radical scavenging activity and reducing power ability (RPA) of *C. procera* root aqueous extracts.

The DPPH is a deep purple colored stable free radical containing an odd electron responsible for absorbance at 540 nm. On acceptance of an electron by an antioxidant compound, the DPPH decolorizes. Aqueous extract of *C. procera* root exhibited a significant dose dependent decolorization of DPPH solution; that is, decrease in absorbance at 540 nm indicates increase in percentage inhibition activity (Figure 3). A strong positive correlation (*r* ^2^ = 0.756) between reducing power and radical scavenging activity of the test extract (Figure 6) was observed which indicated the hydrogen donating potential of the extract. The relationship between both the assays therefore signifies the intrinsic ability of the *C. procera* root aqueous extract to donate the hydrogen atom or electrons and thereby produce antioxidant effect.

Although iron is vital for life, it can be toxic when it is present in excess [45]. The toxic effects of free iron are substantiated by its ability to catalyze via Fenton reaction the generation of damaging reactive free radicals [46]. Iron overload may stimulate lipid peroxidation and formation of hydroxyl radicals with subsequent tissue and organ damage. Lipid peroxidation catalyzed by iron results in the formation of peroxyl radicals (ROO^•^) which finally produces malondialdehyde (MDA). MDA can react with DNA bases to form adducts, which were seen elevated in different pathological conditions especially in cancer [47, 48]. Due to the effective sequestration of iron by the various metal-binding proteins, the cells contain only the negligible amounts of “free catalytic iron.” To avoid the harmful effect of free iron, its proper chelation is of key importance [4, 41, 49]. Study revealed appreciable iron chelating property in the test extract (Figure 4). Root extract has the capability to inhibit the formation of ferrozine—Fe_2_^+^ complex which was measured by the decrease in absorbance at 562 nm.

The process of lipid peroxidation has been associated with damage in cell membranes and propagated through free radical chain reaction [50, 51]. Under normal conditions, incubation of brain, liver, and kidney homogenates in the presence of FeCl_2_ causes a significant increase in lipid peroxidation. However, the presence of *C. procera* root aqueous extract in the reaction mixture was found to inhibit the process of lipid peroxidation substantially during the study (Figure 5). The appreciable inhibition of lipid peroxidation at higher test concentration in liver homogenates by test extract compared to that of brain and kidney homogenates could be attributed to the presence of glutathione and other antioxidants in hepatic cells, while brain cells rely on surrounding astrocyte cells to provide usable glutathione precursors and also limited access to the bulk of antioxidants produced by the body [52]. High lipid content and lower endogenous antioxidant components could also be ascribed to comparatively lower lipid peroxidation inhibition exhibited by test sample in brain and kidney homogenates.

**Figure 7:**
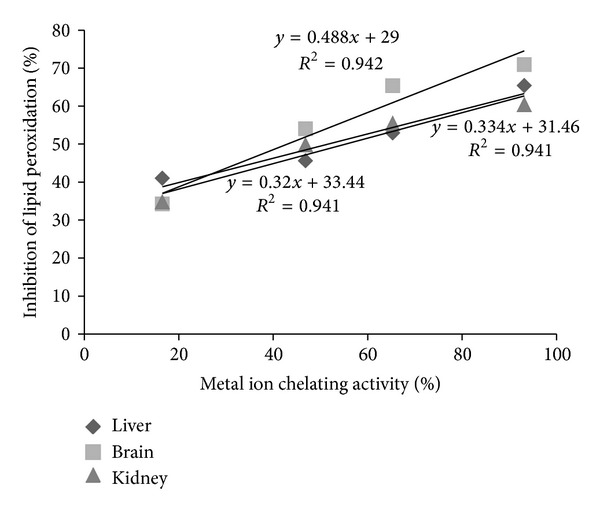
Relationship between metal ion chelating activity and % LPOI in liver, brain, and kidney tissues of albino Wistar rats.

The study established that the test extract has potential to inhibit lipid peroxidation *in vitro.* In additions extracts were also found to have considerable metal ion chelating ability. A strong positive correlation (Figure 7) between metal ion chelation ability and lipid peroxidation inhibition activity of *C. procera* root extract in liver (*r* ^2^ = 0.941), kidney (*r* ^2^ = 0.941), and brain (*r* ^2^ = 0.942) tissue homogenates indicates appreciable antioxidant activity in the aqueous root extracts. Metal ion chelating capacity plays a significant role in the antioxidant mechanism since it reduces the concentration of the catalyzing transition metal in lipid peroxidation. The natural compounds which exhibit high lipid peroxidation inhibition potential can replace the synthetic antioxidants which are known to be toxic. 

## 5. Conclusion

The study demonstrated that phytochemicals present in *Calotropis procera* root have considerable potential to chelate metal ions as well as scavenge free radicals generated in the system and thereby neutralize metal ion mediated oxidative damage. Therefore, *C. procera* root could be regarded as a source of future antioxidant compounds of natural origin.

## References

[1] Mishra A, Kumar S, Bhargava A, Sharma B, Pandey AK (2011). Studies on in vitro antioxidant and antistaphylococcal activities of some important medicinal plants. *Cellular and Molecular Biology*.

[2] Pham-Huy LA, He H, Pham-Huy C (2008). Free radicals, antioxidants in disease and health. *International Journal of Biomedical Science*.

[3] Willcox JK, Ash SL, Catignani GL (2004). Antioxidants and prevention of chronic disease. *Critical Reviews in Food Science and Nutrition*.

[4] Kumar S, Pandey AK (2012). Antioxidant, lipo-protective and antibacterial activities of phytoconstituents present in *Solanum xanthocarpum* root. *International Review of Biophysical Chemistry*.

[5] Pandey AK, Mishra AK, Mishra A, Kumar S, Chandra A (2010). Therapeutic potential of *C. zeylanicum* extracts: an antifungal and antioxidant perspective. *International Journal of Biological and Medical Research*.

[6] Terao J, Piskula MK, Rice-Evans CA, Packer L (1997). Flavonoids as inhibitors of lipid peroxidation in membranes. *Flavonoids in Health and Disease*.

[7] Mishra AK, Mishra A, Bhargava A, Pandey AK (2008). Antimicrobial activity of essential oils from the leaves of *Cinnamomum* spp. *National Academy Science Letters*.

[8] Kumar S, Sharma UK, Sharma AK, Pandey AK (2012). Protective efficacy of *Solanum xanthocarpum* root extracts against free radical damage: phytochemical analysis and antioxidant effect. *Cellular and Molecular Biology*.

[9] Meena AK, Yadav A, Rao MM (2011). Ayurvedic uses and pharmacological activities of *Calotropis procera* Linn. *Asian Journal of Traditional Medicines*.

[10] Larhsini M, Bousaid M, Lazrek HB, Jana M, Amarouch H (1997). Evaluation of antifungal and molluscicidal properties of extracts of *Calotropis procera*. *Fitoterapia*.

[11] Setty SR, Quereshi AA, Viswanath Swamy AHM (2007). Hepatoprotective activity of *Calotropis procera* flowers against paracetamol-induced hepatic injury in rats. *Fitoterapia*.

[12] Mohsin A, Shah AH, Alaha MA, Tariqi MO, Ageel AM (1989). Analgesic, antipyretic activity and phytochemical screening of some plants used in traditional Arab system of medicine. *Fitoterapia*.

[13] Basak SK, Bhaumik A, Mohanta A, Singhal P (2009). Ocular toxicity by latex of *Calotropis procera* (Sodom apple). *Indian Journal of Ophthalmology*.

[14] Dewan S, Sangraula H, Kumar VL (2000). Preliminary studies on the analgesic activity of latex of *Calotropris procera*. *Journal of Ethnopharmacology*.

[15] Mascolo N, Sharma R, Jain SC, Capasso F (1988). Ethnopharmacology of *Calotropis procera* flowers. *Journal of Ethnopharmacology*.

[16] Soares PM, Lima SR, Matos SG (2005). Antinociceptive activity of *Calotropis procera* latex in mice. *Journal of Ethnopharmacology*.

[17] Sharma P, Sharma JD (2000). In-vitro schizonticidal screening of *Calotropis procera*. *Fitoterapia*.

[18] Bhaskar VH, Ajay SS (2009). Antimicrobial activity of *Calotropis procera* seeds. *Asian Journal of Chemistry*.

[19] Mishra AK, Mishra A, Kehri HK, Sharma B, Pandey AK (2009). Inhibitory activity of Indian spice plant *Cinnamomum zeylanicum* extracts against *Alternaria solani* and *Curvularia lunata*, the pathogenic dematiaceous moulds. *Annals of Clinical Microbiology and Antimicrobials*.

[20] Pandey AK (2007). Anti-staphylococcal activity of a pan-tropical aggressive and obnoxious weed *Parthenium histerophorus*: an in vitro study. *National Academy Science Letters*.

[21] Trease GE, Evans WC (1997). *Pharmacognosy*.

[22] Sadasivam S, Manickam A (1996). *Biochemical Methods*.

[23] Singh RP, Murthy KNC, Jayaprakasha GK (2002). Studies on the antioxidant activity of pomegranate (*Punica granatum*) peel and seed extracts using *in vitro* models. *Journal of Agricultural and Food Chemistry*.

[24] Chang C-C, Yang M-H, Wen H-M, Chern J-C (2002). Estimation of total flavonoid content in propolis by two complementary colometric methods. *Journal of Food and Drug Analysis*.

[25] Oyaizu M (1986). Studies on products of browning reactions: antioxidative activities of products of browning reaction prepared from glucosamine. *Japanese Journal of Nutrition*.

[26] Mishra A, Sharma AK, Kumar S, Saxena AK, Pandey AK (2013). *Bauhinia variegata* leaf extracts exhibit considerable antibacterial, antioxidant and anticancer activities. *BioMed Research International*.

[27] Dinis TCP, Madeira VMC, Almeida LM (1994). Action of phenolic derivatives (acetaminophen, salicylate, and 5-aminosalicylate) as inhibitors of membrane lipid peroxidation and as peroxyl radical scavengers. *Archives of Biochemistry and Biophysics*.

[28] Jayaprakasha GK, Girennavar B, Patil BS (2008). Radical scavenging activities of Rio Red grapefruits and sour orange fruit extracts in different *in vitro* model systems. *Bioresource Technology*.

[29] Halliwell B, Gutteridge JNC, Halliwell B, Gsutteridge JMC (1999). Mechanism of damage of cellular targets by oxidative stress: lipid peroxidation. *Free Radicals in Biology and Medicine*.

[30] Gupta M, Shaw BP, Mukherjee A (2010). A new glycosidic flavonoid from *Jwarhar mahakashay* (antipyretic) ayurvedic preparation. *International Journal of Ayurveda Research*.

[31] Sakakibara H, Honda Y, Nakagawa S, Ashida H, Kanazawa K (2003). Simultaneous determination of all polyphenols in vegetables, fruits, and teas. *Journal of Agricultural and Food Chemistry*.

[32] Tripoli E, Guardia ML, Giammanco S, Majo DD, Giammanco M (2007). Citrus flavonoids: molecular structure, biological activity and nutritional properties: a review. *Food Chemistry*.

[33] Robards K, Prenzler PD, Tucker G, Swatsitang P, Glover W (1999). Phenolic compounds and their role in oxidative processes in fruits. *Food Chemistry*.

[34] Hotta H, Nagano S, Ueda M, Tsujino Y, Koyama J, Osakai T (2002). Higher radical scavenging activities of polyphenolic antioxidants can be ascribed to chemical reactions following their oxidation. *Biochimica et Biophysica Acta*.

[35] Govindarajan R, Vijayakumar M, Pushpangadan P (2005). Antioxidant approach to disease management and the role of ‘Rasayana’ herbs of Ayurveda. *Journal of Ethnopharmacology*.

[36] Heim KE, Tagliaferro AR, Bobilya DJ (2002). Flavonoid antioxidants: chemistry, metabolism and structure-activity relationships. *The Journal of Nutritional Biochemistry*.

[37] Cushnie TPT, Lamb AJ (2005). Antimicrobial activity of flavonoids. *International Journal of Antimicrobial Agents*.

[38] Mishra AK, Singh BK, Pandey AK (2010). *In vitro*-antibacterial activity and phytochemical profiles of *Cinnamomum tamala* (Tejpat) leaf extracts and oil. *Reviews in Infection*.

[39] Maurya A, Chauhan P, Mishra A, Pandey AK (2012). Surface functionalization of TiO_2_ with plant extracts and their combined antimicrobial activities against *E. faecalis* and *E. coli*. *Journal of Research Updates in Polymer Science*.

[40] Kumar VL, Padhy BM (2011). Protective effect of aqueous suspension of dried latex of *Calotropis procera* against oxidative stress and renal damage in diabetic rats. *Biocell*.

[41] Kumar S, Mishra A, Pandey AK (2013). Antioxidant mediated protective effect of *Parthenium hysterophorus* against oxidative damage using *in vitro* models. *BMC Complementary and Alternative Medicine*.

[42] Tanaka M, Kuie CW, Nagashima Y, Taguchi T (1988). Application of antioxidative Maillard reaction products from histidine and glucose to sardine products. *Nippon Suisan Gakk*.

[43] Pandey AK, Mishra AK, Mishra A (2012). Antifungal and antioxidative potential of oil and extracts derived from leaves of indian spice plant *Cinnamomum tamala*. *Cellular and Molecular Biology*.

[44] Gordon MH, Hudson BJ (1990). The mechanism of antioxidant action in vitro. *Food Antioxidants*.

[45] Lee DW, Andersen JK, Kaur D (2006). Iron dysregulation and neurodegeneration: the molecular connection. *Molecular Interventions*.

[46] Ganz T (2003). Hepcidin, a key regulator of iron metabolism and mediator of anemia of inflammation. *Blood*.

[47] Marnett LJ (1999). Lipid peroxidation—DNA damage by malondialdehyde. *Mutation Research*.

[48] Wang Z, Rossman TG, Chang T (1996). The carcinogenicity of arsenic. *Toxicology of Metals*.

[49] Kell DB (2009). Iron behaving badly: inappropriate iron chelation as a major contributor to the aetiology of vascular and other progressive inflammatory and degenerative diseases. *BMC Medical Genomics*.

[50] Halliwell B (1989). Protection against tissue damage in vivo by desferrioxamine: what is its mechanism of action?. *Free Radical Biology and Medicine*.

[51] Kumar S, Pandey AK (2013). Phenolic content, reducing power and membrane protective activities of *Solanum xanthocarpum* root extracts. *Vegetos*.

[52] Perry SW, Norman JP, Litzburg A, Gelbard HA (2004). Antioxidants are required during the early critical period, but not later, for neuronal survival. *Journal of Neuroscience Research*.

